# Data supporting the results of the characterization of the phases and structures appearing during the synthesis process of Ba_0.5_Sr_1.5_Zn_2-x_Ni_x_Fe_12_O_22_ by auto-combustion

**DOI:** 10.1016/j.dib.2020.105803

**Published:** 2020-06-02

**Authors:** Tatyana Koutzarova, Svetoslav Kolev, Kiril Krezhov, Borislava Georgieva, Chavdar Ghelev, Daniela Kovacheva, Benedicte Vertruyen, Raphael Closset, Lan Maria Tran, Michal Babij, Andrzej J. Zaleski

**Affiliations:** a Institute of Electronics, Bulgarian Academy of Sciences, 72 Tsarigradsko Chaussee, 1784 Sofia, Bulgaria; b Institute of General and Inorganic Chemistry, Bulgarian Academy of Sciences, Acad. Georgi Bonchev Str., bld. 11, 1113 Sofia, Bulgaria; c Greenmat, Chemistry Department, University of Liege, 11 allée du 6 août, 4000 Liège, Belgium; d Institute of Low Temperature and Structure Research, Polish Academy of Sciences, Ul. Okólna 2, 50-422 Wroclaw, Poland

**Keywords:** Y type hexaferrite, Powder annealing, Crystalline size, Cation ratio, Auto-combustion technique

## Abstract

The data presented has to do with identifying the various phases arising during the synthesis of the Y-type hexaferrite series Ba_0.5_Sr_1.5_Zn_2-x_Ni_x_Fe_12_O_22_ by auto-combustion that we deem important for their microstructural and magnetic properties. The data and the related analyses support the research paper “Ni-substitution effect on the properties of Ba_0.5_Sr_1.5_Zn_2-x_Ni_x_Fe_12_O_22_ powders” [1]. Thus, the parameters are presented of the phases appearing after auto-combustion and after the initial annealing at 800 °C, namely, crystal cell and crystallite size. Also, additional data are provided obtained by EDS concerning the Ba:Sr:Zn:Ni:Fe ratio in Ba_0.5_Sr_1.5_Zn_2-x_Ni_x_Fe_12_O_22_ (x = 0.8, 1, 1.5) samples synthesized at 1170 °C for 10 h. The data can be used as a reference in establishing how the phases distinguished during the initial process of auto-combustion affect the Ba_0.5_Sr_1.5_Zn_2-x_Ni_x_Fe_12_O_22_ powders, which are candidates for room-temperature multiferroic materials. The data have not been published previously and are made available to permit critical or further analyses.

Specifications Table

**Table:** 

**Subject**	Materials Science, Electronic, Optical and Magnetic Materials
**Specific subject area**	Multiferroic Materials, Hexaferrites, Sol-Gel Auto-Combustion
**Type of data**	Tables Figures Text file
**How data were acquired**	X-ray diffraction (XRD) measurements performed using a Brucker D8 diffractometer Scanning electron microscopy and energy dispersive X-ray spectroscopy (FEI XL30 FEG-ESEM, Bruker Quantax EDS coupled to ESEM)
**Data format**	Raw Analyzed Filtered
**Parameters for data collection**	The characterization was implemented by X-ray diffraction (XRD) carried out by a Brucker D8 diffractometer (40 kV, 30 mA) controlled by DIFFRACTPLUS software in Bragg-Brentano reflection geometry with Cu-Kα radiation (λ = 1.5418 Å); and by scanning electron microscopy and energy dispersive X-ray spectroscopy (FEI XL30 FEG-ESEM, Bruker Quantax EDS coupled to ESEM).
**Description of data collection**	The XRD experiments were conducted on powder samples. The percentage, crystal cell parameters and crystallite size of the phases were determined from X-ray diffractograms. The EDS analyses were performed on four points on polished cross-sections of bulk samples (pellets) in view of finding the Ba:Sr:Zn:Ni:Fe ratio.
**Data source location**	Institution: Institute of Electronics, Bulgarian Academy of Sciences City: Sofia Country: Bulgaria Institution: Institute of General and Inorganic Chemistry City: Sofia Country: Bulgaria Institution: Greenmat, Chemistry Department, University of Liege, City: Liège Country: Belgium
**Data accessibility**	With the article
**Related research article**	T. Koutzarova, S. Kolev, K. Krezhov, B. Georgieva, Ch. Ghelev, D. Kovacheva, B. Vertruyen, R. Closset, L. M. Tran, M. Babij, A. J. Zaleski, Ni-substitution effect on the properties of Ba_0.5_Sr_1.5_Zn_2-x_Ni_x_Fe_12_O_22_ powders, J. Magn. Magn. Mater. doi: 10.1016/j.jmmm.2020.166725

**Value of the Data**

• The data distinguishes the intermediate phases appearing during the synthesis of a Y-type hexaferrite (Ba_0.5_Sr_1.5_Zn_2-x_Ni_x_Fe_12_O_22_) by auto-combustion;
• The EDS analysis yields reliable information on the chemical composition at different points on bulk Ba_0.5_Sr_1.5_Zn_2-x_Ni_x_Fe_12_O_22_ samples;
• The data could be useful in identifying the phases and their effect during the synthesis of other hexaferrites;
• The data supplies important additional information to the related research article.

## 1 Data description

**Fig. 1:**
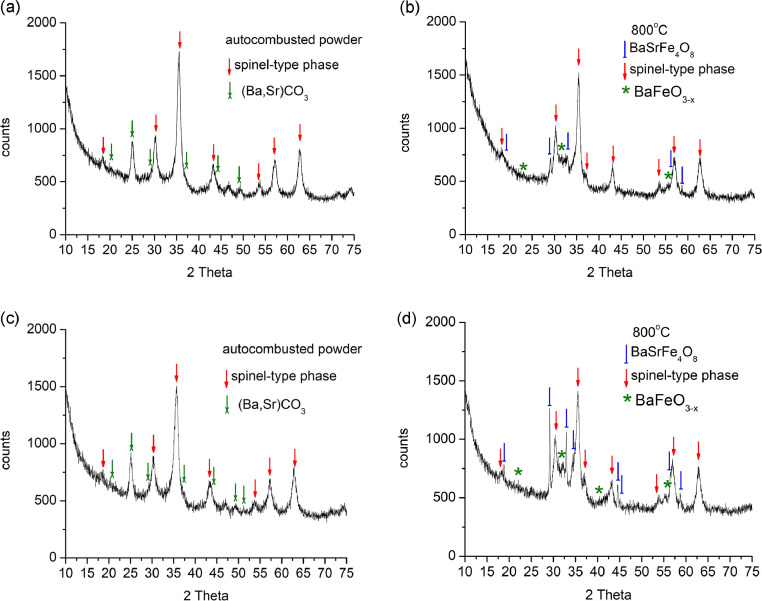
XRD-patterns of auto-combusted powders (a, c) and powder annealed at 800 °С for three hours (b, d) for samples x = 1 (a, b), and 1.5 (c, d).

The data and analyses included here corroborate the results of and the conclusions drawn from the study of Ba_0.5_Sr_1.5_Zn_2-x_Ni_x_Fe_12_O_22_ (x = 0.8, 1, and 1.5) hexaferrites synthesized by sol-gel auto-combustion [1]. The XRD analysis was employed to distinguish between the phases formed during the consecutive steps of the synthesis of Ba_0.5_Sr_1.5_Zn_2-x_Ni_x_Fe_12_O_22_ (x = 0.8, 1, and 1.5). Fig. 1 shows the XRD patterns of the auto-combusted powders and the powder treated at 800 °С for three hours for samples with x = 1, and 1.5. The corresponding XRD patterns for sample Ba_0.5_Sr_1.5_Zn_1.2_Ni_0.8_Fe_12_O_22_ are presented in Koutzarova et al. [1]. A spinel-type phase (Ni-Zn mixed ferrite (Zn, Ni)Fe_2_O_4_) and (Ba, Sr)CO_3_ were observed in the as-synthesized auto-combustion powders. The XRD-patterns of the powders heat-treated at 800 °С exhibit the peaks of spinel type phase BaFeO_3-x_ and BaSrFe_4_O_8_. Table 1 summarizes the data for the crystal cell parameters, the average crystallite size and the phases’ percentage content derived from the XRD patterns. The original output files for all three samples (x = 0.8, 1, and 1.5) obtained by Topas 4.2 are given in the supplementary file.

**Table 1:** Phase percentage, crystal cell parameters and average crystallite size obtained from the XRD patterns of auto-combusted powders and powder annealed at 800 °С for three hours.

Sample	Spinel	(Ba,Sr)CO_3_	(Ba,Sr)FeO_3-x_	BaSrFe_4_O_8_
Ba_0.5_Sr_1.5_Zn_1.2_Ni_0.8_Fe_12_O_22_ auto-combusted powder percentage *a* *b* *c* crystallite size	87% 8.378(6) Å 10 nm	13% 5.099(4) Å 8.624(9) Å 6.098(4) Å 19 nm		
Ba_0.5_Sr_1.5_Zn_1.2_Ni_0.8_Fe_12_O_22_ annealed at 800°С percentage *a* *b* *c* crystallite size	65% 8.395(5) Å 11 nm		25% 5.664(1) Å 21.681(4) Å 5 nm (fix)	10% 5.435(5) Å 8.055(5) Å 79 nm
Ba_0.5_Sr_1.5_ZnNiFe_12_O_22_ auto-combusted powder percentage *a* *b* *c* crystallite size	87% 8.371(1) Å 11 nm	13% 5.098(2) Å 8.621(8) Å 6.105(5) Å 19 nm		
Ba_0.5_Sr_1.5_ZnNiFe_12_O_22_ annealed at 800°С percentage *a* *b* *c* crystallite size	68% 8.385(1) Å 12 nm		27% 5.685(4) Å 21.860(3) Å 5 nm (fix)	5% 5.446(2) Å 8.071(6) Å 24 nm
Ba_0.5_Sr_1.5_Zn_0.5_Ni_1.5_Fe_12_O_22_ auto-combusted powder percentage *a* *b* *c* crystallite size	88% 8.357(8) Å 9 nm	12% 5.101(0) Å 8.601(6) Å 6.097(6) Å 21 nm		
Ba_0.5_Sr_1.5_Zn_0.5_Ni_1.5_Fe_12_O_22_ annealed at 800°С percentage *a* *b* *c* crystallite size	67% 8.368(1) Å 10 nm		27% 5.659(0) Å 21.740(4) Å 5 nm (fix)	6% 5.437(0) Å 8.052(9) Å 79 nm

**Fig. 2:**
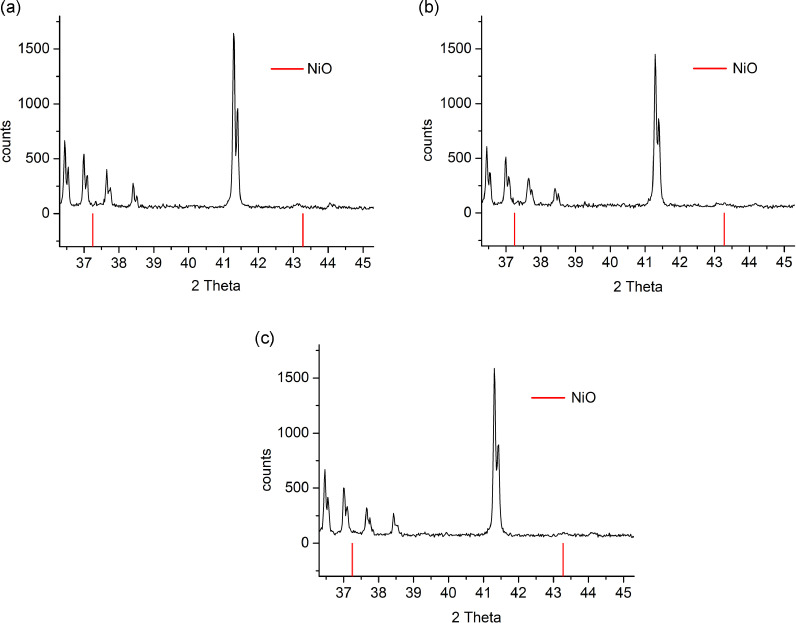
XRD-patterns of (a) Ba_0.5_Sr_1.5_Zn_1.2_Ni_0.8_Fe_12_O_22_, (b) Ba_0.5_Sr_1.5_ZnNiFe_12_O_22_ and (c) Ba_0.5_Sr_1.5_Zn_0.5_Ni_1.5_Fe_12_O_22_ in the 2-theta range 37.3° – 45.3°. The expected peak positions of NiO (PDF number 00–047–1049) are marked in red.

Fig. 2 displays XRD-patterns in the 2-theta range 37.3° – 45.3° of Ba_0.5_Sr_1.5_Zn_1.2_Ni_0.8_Fe_12_O_22_, Ba_0.5_Sr_1.5_ZnNiFe_12_O_22_ and Ba_0.5_Sr_1.5_Zn_0.5_Ni_1.5_Fe_12_O_22_ treated at 1170 °C. No signs were observed of nickel spinel ferrite decomposition to NiO during the Y-type hexaferrite formation process [2].

The statistical data yielded by the analysis of the four points of EDX spectrum of polished cross-sections of the bulk samples of Ba_0.5_Sr_1.5_Zn_1.2_Ni_0.8_Fe_12_O_22_, Ba_0.5_Sr_1.5_ZnNiFe_12_O_22_ and Ba_0.5_Sr_1.5_Zn_0.5_Ni_1.5_Fe_12_O_22_ are given in Table 2.

**Table 2:** The Ba:Sr:Zn:Ni:Fe ratio estimated from the EDX analyses of Ba_0.5_Sr_1.5_Zn_2-x_N_ix_Fe_12_O_22_ (x = 0.8, 1, 1.5) samples at the four points on polished cross-sections surface.

Sample	Ba_0.5_Sr_1.5_Zn_1.2_Ni_0.8_Fe_12_O_22_	Ba_0.5_Sr_1.5_ZnNiFe_12_O_22_	Ba_0.5_Sr_1.5_Zn_0.5_Ni_1.5_Fe_12_O_22_
	Ba:Sr:Zn:Ni:Fe	Ba:Sr:Zn:Ni:Fe	Ba:Sr:Zn:Ni:Fe
Point 1	0.5:1.5:1.1:0.8:12.3	0.5:1.7:1.7:1.7:16.7	0.5:1.5:0.5:1.4:12.9
Point 2	0.5:1.6:1.2:0.7:12.4	0.5:1.5:1.8:1.5:16.6	0.5:1.5:0.5:1.3:12.0
Point 3	0.5:1.5:1.1:0.8:12.4	0.5:1.7:1.1:1.0:12.3	0.5:1.7:0.5:1.3:12.6
Point 4	0.5:1.5:1.0:0.8:12.2	0.5:1.6:2.2:2.2:15.1	0.5:1.6:0.6:1.4:12.9

## 2 Experimental design, materials, and methods

Polycrystalline samples of Ba_0.5_Sr_1.5_Zn_2-x_Ni_x_Fe_12_O_22_ (x = 0.8, 1, and 1.5) were fabricated by a modified citric acid sol-gel auto-combustion using stoichiometric amounts of the precursors; a detailed description of the sample preparation methodology is given in [1]. In brief, the powders produced after the auto-combustion process were annealed at 800 °С for three hours. All powders were subjected to homogenization by vibrating ball milling; then the resulting powders were pressed at 7 MPa to bulk pellets with a diameter of 16 mm. The pellets were heat-treated at 1170 °С in air for 10 h to obtain the Ba_0.5_Sr_1.5_Zn_2-x_Ni_x_Fe_12_O_22_ compositions with *x* = 0.8, 1, and 1.5. Subsequently, the bulk pellets were cut and polished for microscopic studies.

## Declaration of Competing Interest

The authors declare that they have no known competing financial interests or personal relationships which have, or could be perceived to have, influenced the work reported in this article.

## Appendix Supplementary materials

Image, application 1
